# Machine Learning Applications for Differentiation of Glioma from Brain Metastasis—A Systematic Review

**DOI:** 10.3390/cancers14061369

**Published:** 2022-03-08

**Authors:** Leon Jekel, Waverly R. Brim, Marc von Reppert, Lawrence Staib, Gabriel Cassinelli Petersen, Sara Merkaj, Harry Subramanian, Tal Zeevi, Seyedmehdi Payabvash, Khaled Bousabarah, MingDe Lin, Jin Cui, Alexandria Brackett, Amit Mahajan, Antonio Omuro, Michele H. Johnson, Veronica L. Chiang, Ajay Malhotra, Björn Scheffler, Mariam S. Aboian

**Affiliations:** 1 Department of Radiology and Biomedical Imaging, Yale School of Medicine, 333 Cedar Street, P.O. Box 208042, New Haven, CT 06510, USA; leon.jekel@yale.edu (L.J.); waverly.brim@yale.edu (W.R.B.); marc.vonreppert@yale.edu (M.v.R.); lawrence.staib@yale.edu (L.S.); gabriel.cassinellipetersen@yale.edu (G.C.P.); sara.merkaj@yale.edu (S.M.); harry.subramanian@yale.edu (H.S.); tal.zeevi@yale.edu (T.Z.); sam.payabvash@yale.edu (S.P.); mingde.lin@yale.edu (M.L.); jcui527@gmail.com (J.C.); amit.mahajan@yale.edu (A.M.); michele.h.johnson@yale.edu (M.H.J.); ajay.malhotra@yale.edu (A.M.); 2 DKFZ Division of Translational Neurooncology at the WTZ, German Cancer Consortium, DKTK Partner Site, University Hospital Essen, 45147 Essen, Germany; b.scheffler@dkfz-heidelberg.de; 3 German Cancer Research Center, 69120 Heidelberg, Germany; 4 Department of Computer Science, The Johns Hopkins Whiting School of Engineering, 3400 North Charles Street, Baltimore, MD 21218, USA; 5 Visage Imaging GmbH, Lepsiusstraße 70, 12163 Berlin, Germany; kbousabarah@visageimaging.com; 6 Visage Imaging Inc., 12625 High Bluff Dr, San Diego, CA 92130, USA; 7 Harvey Cushing/John Hay Whitney Medical Library, Yale School of Medicine, 333 Cedar Street, New Haven, CT 06510, USA; alexandria.brackett@yale.edu; 8 Department of Neurology, Yale School of Medicine, 15 York St Ste LCI702, New Haven, CT 06510, USA; antonio.omuro@yale.edu; 9 Department of Neurosurgery, Yale University School of Medicine, New Haven, CT 06510, USA; veronica.chiang@yale.edu; 10 Department of Therapeutic Radiology, Yale University School of Medicine, New Haven, CT 06510, USA

**Keywords:** systematic review, glioma, glioblastoma, brain metastasis, machine learning, artificial intelligence, reporting quality assessment

## Abstract

**Simple Summary:**

We present a systematic review of published reports on machine learning (ML) applications for the differentiation of gliomas from brain metastases by summarizing study characteristics, strengths, and pitfalls. Based on these findings, we present recommendations for future research in this field.

**Abstract:**

Glioma and brain metastasis can be difficult to distinguish on conventional magnetic resonance imaging (MRI) due to the similarity of imaging features in specific clinical circumstances. Multiple studies have investigated the use of machine learning (ML) models for non-invasive differentiation of glioma from brain metastasis. Many of the studies report promising classification results, however, to date, none have been implemented into clinical practice. After a screening of 12,470 studies, we included 29 eligible studies in our systematic review. From each study, we aggregated data on model design, development, and best classifiers, as well as quality of reporting according to the TRIPOD statement. In a subset of eligible studies, we conducted a meta-analysis of the reported AUC. It was found that data predominantly originated from single-center institutions (n = 25/29) and only two studies performed external validation. The median TRIPOD adherence was 0.48, indicating insufficient quality of reporting among surveyed studies. Our findings illustrate that despite promising classification results, reliable model assessment is limited by poor reporting of study design and lack of algorithm validation and generalizability. Therefore, adherence to quality guidelines and validation on outside datasets is critical for the clinical translation of ML for the differentiation of glioma and brain metastasis.

## 1. Introduction

Gliomas and brain metastases are the most common brain malignancies and account for a substantial proportion of cancer-related mortality [1]. Brain metastases (BM) occur in 2% of patients with cancer at the point of diagnosis, appear in 12.1% of patients with metastatic disease to any site [2], and occur with the site of primary tumor being unknown in up to 15% of patients at first presentation with cerebral metastasis [3]. Gliomas make up more than 30% of the overall tumors of the central nervous system (CNS) and account for 81% of total CNS malignancies [4]. Clinical management of gliomas and brain metastases varies immensely, thus requiring differential diagnosis early in the course of evaluation.

Magnetic resonance imaging (MRI) is currently regarded as the reference standard for evaluation of cerebral malignancies and their effects on the brain, therapeutic response, and overall disease progression [5]. Classic metastatic disease to the brain can be easily differentiated in clinical practice from gliomas in the setting of multiple metastases and imaging features of defined lesion borders and prominent surrounding edema. On the contrary, solitary parenchymal brain metastases, which are seen in approximately 30% of patients with CNS metastasis [5,6,7], can mimic the appearance of higher-grade gliomas, in particular glioblastoma (GBM), and complicate accurate diagnosis, especially when the primary site of cancer is unknown upon first discovery of metastasis. In conventional high-resolution MRI, definitive diagnosis of the lesion can be ambiguous. For example, rim-enhancing lesions in contrast-enhanced T1 sequences are most often found to be high-grade gliomas (40%), closely followed by brain metastases (30%) [7]. While recent research has shown added value of advanced MR imaging techniques, such as MR perfusion and spectroscopy, for tumor differentiation, these methods are not always implemented in standard clinical practice, and standard imaging protocols often feature conventional MRI only. 

The increasing volume of medical imaging data and the exponential growth of computational power over the course of the last years has propelled the investigation of machine learning (ML) applications in neuroradiology, especially for tasks that require specialized expertise. ML algorithms can perform complex tasks without explicit programming, but instead by learning analytically through exposure to data with the subsequent ability to model complex associations, e.g., for image segmentation or image interpretation [8]. Several studies have developed predictive ML models for increased diagnostic performance in differentiation of cerebral metastatic disease from glioma, however, incorporation of these algorithms into clinical practice has not been achieved yet, as per the ACR Data Science Institute AI Central [9].

We present a literature review, as well as summary reports on average model performance to identify the most promising approaches reported in the current body of literature. We aim to detect shared pitfalls to clinical implementation in the field of differentiation of brain metastases and gliomas. Through our assessment of quality of reporting, we aim to systematically analyze the shortcomings that are common in the literature and, ultimately, formulate recommendations for researchers engaging in this growing field. We aim to identify, to which degree the opacity of reporting and lack of standardization in algorithm development prevail in the literature, which, as pointed out previously [10,11], are detrimental to clinical translation and FDA clearance of ML models.

## 2. Materials and Methods

### 2.1. Database Search

**Figure 1:**
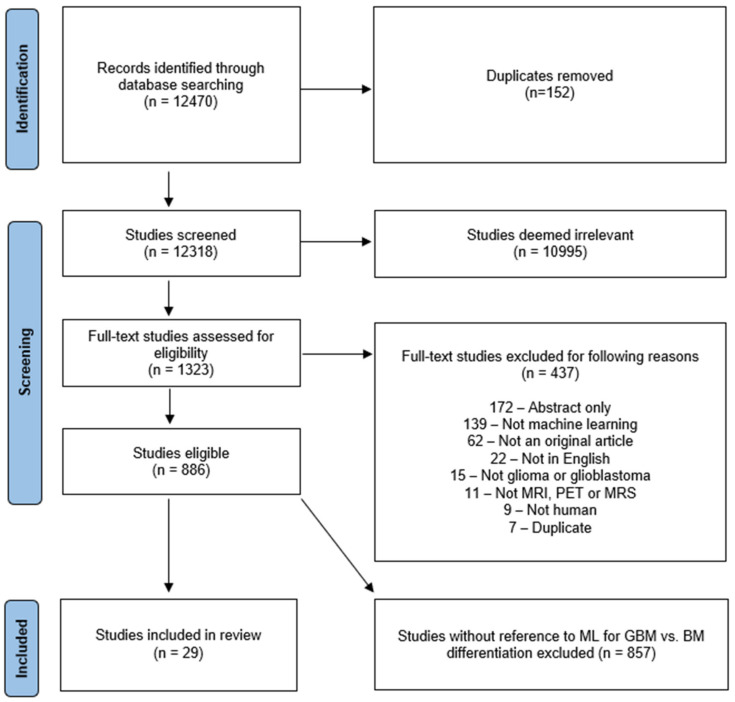
Characterization of search strategy using PRISMA. This flowchart represents the search and screening workflow and the eligibility criteria applied to the studies. BM = brain metastasis.

This systematic review was registered with the International Prospective Register of Systematic Reviews (PROSPERO) under the registration number CRD42020209938 and conducted according to the guidelines of the PRISMA (Preferred Reporting Items for Systematic Reviews and Meta-Analyses of Diagnostic Test Accuracy) [12] statement. To collect all relevant original research on the applications of AI in neuro-oncology, database searches of Ovid Embase, OVID MEDLINE, Cochrane trials (CENTRAL), and Web of Science—Core Collection were conducted by a clinical librarian (A.B.) in September 2020, January 2021, and September 2021, respectively. The search strategy included both keywords and controlled vocabulary combining the terms for: “artificial intelligence”, “machine learning”, “deep learning”, “radiomics”, “magnetic resonance imaging”, “glioma”, as well as related terms. The search strategy was independently reviewed by a second institutional librarian. All publications identified by the search were subjected to a screening in Covidence software (Veritas Health Innovation Ltd. Melbourne, Australia). The search identified 12,470 candidate articles (Figure 1). 152 duplicates were removed and screening of the remaining 12,318 articles was conducted by a neuroradiology attending (M.A.), radiology resident (H.S.) and three graduate students (L.J., W.B. and M.v.R.). The board-certified neuroradiology attending (M.A.) resolved ambiguous screening conflicts. The abstract review further excluded 10,995 articles that lacked pertinence to neuro-oncology and ML. A total of 1323 articles were reviewed at full-text level and evaluated for eligibility for inclusion in the review. For reviewer-independent uniformity in screening, 8 exclusion criteria were predefined: (1) Abstract-only; (2) No application of ML reported; (3) Not an original article; (4) Not published in English; (5) No investigation of glioma/glioblastoma; (6) Unrelated to MRI, magnetic resonance spectroscopy (MRS), and positron emission tomography (PET) imaging; (7) No human research subjects; (8) Duplicates. Due to fulfilment of at least one of those criteria, 437 additional studies were excluded. 886 eligible full text studies were then reviewed by either a radiology resident (H.S.) or a graduate student (L.J., W.B. and G.C.P.), in addition to a second review by the board-certified neuroradiologist (M.A.). Twenty-nine studies that were identified to specifically investigate the differentiation of glioma from brain metastasis were then analyzed in the present study. The search strategy is provided in the Supplementary Materials (Figure S1).

### 2.2. Data Extraction and Aggregation

The data extraction was performed independently by two reviewers (L.J., W.B.) using predefined tables in Microsoft Excel. Disagreements were discussed in regular team meetings and were resolved with a supervising neuroradiology attending (M.A.) until consensus was reached. Data points compiled in this study include: article characteristics (title, author, publication year), data characteristics (data source, dataset size, types and number of tumors for training/testing/validation, model validation technique), class balancing (ratio of glioma to brain metastases), model characteristics (best performing ML classifier, classification task, type of features and imaging sequences used for classification, outcome measures for classifier performance) (Table 1), and reporting characteristics. Whenever referred to in this study, internal validation describes measures for assessment of quality and robustness of the model and its ability to predict outcomes on unseen data. Some studies report three-way partitioning of their dataset into training, validation, and testing sets. In this context, validation data serves to mathematically optimize and finetune model hyperparameters and should not be confused with overall internal validation.

### 2.3. Descriptive Statistics and Performance Evaluation Metrics

All performance metrics (accuracy, sensitivity, specificity, area under the receiver operating characteristics curve (AUC)) for the best performing classifier from each study were aggregated, tabulated, and displayed using GraphPad Prism version 8.3.4 (GraphPad Software, San Diego, CA, USA, www.graphpad.com (accessed on 1 March 2022)). As benchmark for definition of the best performing classifier from a study, we referred strictly to the AUC. When AUC was not reported, classification accuracy was considered for the classifier ranking. The performance metrics of the best classifiers were pooled; however, only algorithms with pairwise classification between gliomas and brain metastases were considered for this synthesis. The remaining studies presented multiclass approaches and did not provide dichotomized model discrimination measures. Internally validated and externally validated classification results were reported separately.

### 2.4. Assessment of Quality of Reporting

The present systematic review also includes a thorough assessment of reporting quality. We conducted a TRIPOD [41] adherence evaluation for model development studies, a reporting guideline with 22 main items and 65 adherence elements in total. The TRIPOD scores were evaluated by two reviewers and, in case of disagreement, consensus by discussion was made. For calculation of TRIPOD adherence scores, we followed the appraisal guidelines [42]. Average TRIPOD adherence score and average degree of item satisfaction, indicated as adherence index (ADI) ranging from 0 to 1, were determined using Microsoft Excel.

### 2.5. Statistical Analysis

Reporting quality was compared in study cohorts published before and during/after 2019. The difference in median TRIPOD adherence score was examined for statistical significance using the Mann-Whitney U test in MedCalc version 20.019 (MedCalc Software bv, Ostend, Belgium; https://www.medcalc.org (accessed on 1 March 2022); 2021). Differences in mean AUC between study subsets were tested for statistical significance via Student’s t-test using GraphPad Prism version 8.3.4. All eligible studies that reported AUC (area under the ROC curve) and standard error of mean (SEM) or 95% confidence intervals (CI), were subjected to a meta-analysis using MedCalc. In one study, SEM was imputed from reported standard deviation (SD) of the mean and sample size. These metrics were subjected to a random effects model. Results of this quantitative analysis were then illustrated in a forest plot [43]. Heterogeneity within the analyzed studies was then examined via the Higgins I^2^-test [44].

## 3. Results

### 3.1. Study Selection

This systematic review identified 29 eligible studies as part of the literature on ML models for differentiation of gliomas from brain metastases (Figure 1). The studies were published between 2008 and 2021. Yamashita et al. [16] presented a predictive model that used a threshold for differentiation of glioma from brain metastasis. This threshold was determined by shape evaluation performed by an unsupervised ML algorithm and was therefore included in this systematic review.

### 3.2. Study Characteristics

#### 3.2.1. Datasets

**Figure 2:**
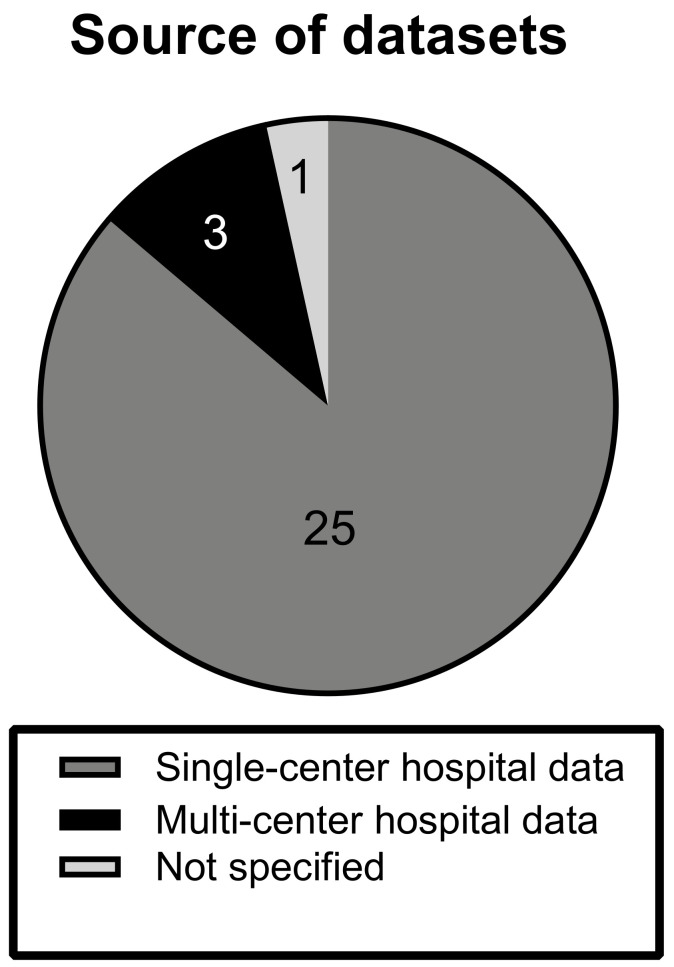
Source of datasets. The chart displays the distribution of types of datasets, from which MRI scans were derived, among the different studies. Note how the majority (89%) of studies trained and validated on single-center data.

The investigated articles predominantly made use of local single-center hospital datasets (n = 25). Three studies were conducted on hospital data from multiple datasets. In one study, the source of data was not specified and is unclear [16] (Figure 2).

#### 3.2.2. Dataset Composition

**Figure 3:**
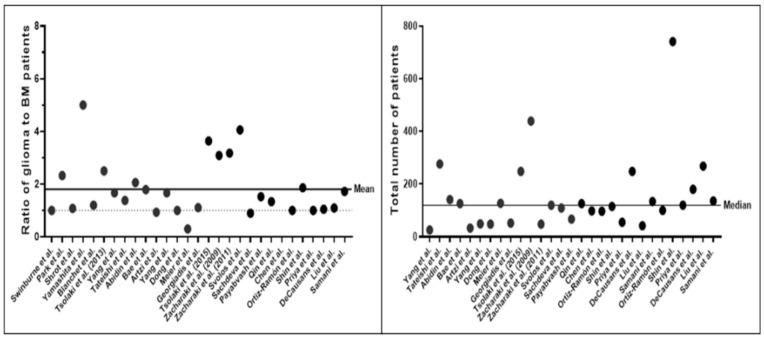
Class distribution of gliomas and brain metastases (**left**) and total number of patient studies (**right**) in each study. The panel on the left-hand side shows the ratio of glioma and brain metastasis patients among the different datasets. The dotted line indicates equal class distribution, i.e. class balance. The right-hand panel indicates the total number of patients across all studies. Note that most studies were trained and validated on datasets with less than 200 patients.

The total numbers of subjects described by the studies were overall small, averaging at 154.10 ± 147.25 (mean ± SD) patients. Deep learning (DL) studies used larger datasets, averaging at 350.25 ± 99.00 (mean ± SD). Datasets were found to exhibit a glioma-to-BM ratio of 1.80 (±1.10):1 (Figure 3). While every study presented a ML model for differentiation between gliomas and brain metastasis, classification tasks and inclusion criteria for subjects varied. Ten studies included further tumor entities, such as atypical meningioma [30] or primary CNS lymphoma [13] as prediction classes. While 15 studies specified inclusion of patients with only solitary brain metastasis, this was not explicitly mentioned in nine studies. Five studies explicitly stated the inclusion of BM patients with multiple lesions. Twenty studies reported the exclusive investigation of GBM. The rest of the studies (n = 9) also included lower-grade and/or atypical glioma patients. Tsolaki et al. [28] performed intra-class subgroup analyses and compared classification accuracy for differentiation of brain metastases against different glioma grades. Artzi et al. [23] and Meier et al. [26] differentiated between multiple types of brain metastases for further subtype analyses of brain tumor etiology.

#### 3.2.3. Imaging Modalities and Features

Different MR sequences were used for tumor measurements and subsequent feature extraction. In 25 out of 29 studies, contrast-enhanced T1 (T1CE) sequences were utilized within the workflow described in the study for delineation of the region of interest (ROI), which is the standard-of-care sequence in brain metastases. Only two studies [19,24] did not derive their ROIs from conventional MR imaging, but via segmentation on diffusion tensor imaging. Imaging features from conventional MRI were employed for classification in 23 studies. Ten studies included diffusion-weighted imaging, and six studies included perfusion-weighted imaging, five of which specified the use of dynamic susceptibility contrast (DSC) imaging, whereas the remaining one provided no further information. Four studies included MR spectroscopy. Multiple studies showed that classification results upon investigation of the peritumoral area (outside of the contrast-enhancing border on T1CE) were superior to those yielded from the intratumoral portion [18,28,30]. Similarly, Samani et al. [40] showed high classification results from exploiting the DTI-derived free water volume fraction from the peritumoral microenvironment. These findings suggest a reflection of the disparate tumor biology and mode of growth, which is of an infiltrative nature in glioma and of an expansile nature in brain metastases.

The imaging features used for classification of gliomas from brain metastases were heterogeneous and included clinical, qualitative, and semantic imaging features, as well as shape features and radiomics features of first, second and higher orders (Figure S3). Second-order, i.e. textural quantitative imaging features, were found to be employed most frequently (n = 12).

#### 3.2.4. Algorithms

We aimed to identify the most common ML algorithms in the investigated body of literature. Most studies (n = 19) evaluated multiple classifiers in their study. Support vector machines (SVM) (n = 21) and k-nearest neighbors algorithms (kNN) (n = 10) were the most frequently investigated classifiers. Deep learning (DL) techniques, such as deep neural networks (DNN), or convolutional neural networks (CNN), were leveraged less (n = 4), but were increasingly represented in more recent publications between 2020 and 2021. The 29 best performing classifiers drawn from each study showed a variety of different algorithms. Among those, SVM (support vector machine) and its variations were again represented the most (n = 9). Lesser used traditional ML algorithms were used in the rest of the studies and were grouped into non-DL neural networks (n = 4), logistic regression (n = 3), tree-based ensemble classifiers (n = 2), namely Random Forest and AdaBoost, k-nearest neighbors (n = 2), and others (n = 2). DL-based algorithms, namely CNN (n = 3) and a DNN (n = 1), were the best reported classifiers in four studies. Algorithm representation among all reported classifiers versus representation among the best performing classifiers is visualized in Figure S4a.

#### 3.2.5. Model Validation

Internal validation measures were reported in every study. Cross-validation, particularly leave-one-out cross validation (LOOCV) (n = 9), was performed in 21 studies. Two studies [31,36] presented a three-way split of their dataset into training, validation, and testing set. External validation sets stem from a geographically distinct location and should ensure that the model generalizes well onto data from foreign populations. This was only conducted in two studies [22,36].

#### 3.2.6. Classification Performance

Classification accuracy (n = 19) was the most reported model evaluation metric. One study [26] exclusively provided a F1-Score, the harmonic mean of precision and recall, of 0.865 without class balancing and 0.326 with class balancing, respectively. We aggregated the internally validated performance metrics from all studies that provided dichotomized models for classification between glioma and brain metastases (n = 26). Two studies provided evaluation metrics for differentiation of brain metastasis from the four individual glioma grades separately. For our data aggregation, we opted to only include the herein reported differentiation between “Grade 4 glioma and brain metastasis”, which more precisely reflects the clinical diagnostic dilemma in differentiating high grade gliomas from solitary brain metastases. Note that the following syntheses do not meet the criteria of a meta-analysis as most studies failed to provide estimates of level of certainty, such as SEM or CI.

**Figure 4:**
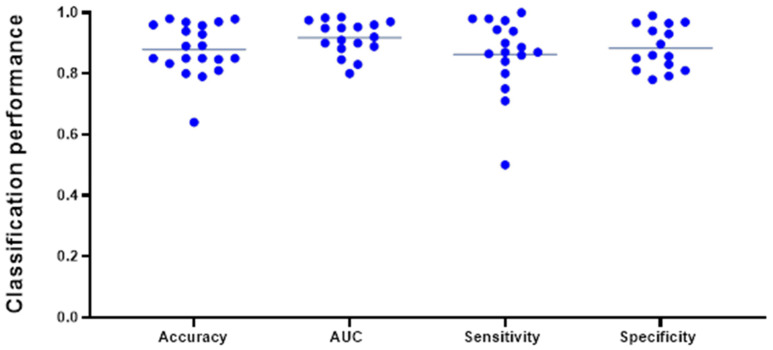
Most frequently reported performance metrics for the best performing classifier from each study included in this data aggregation (n = 26). The lines indicate the mean of the different metrics and reached an overall high level. Note that not all the above-mentioned evaluation metrics were indicated for every classifier and are displayed in different amounts in this plot.

The pooled average of all studies reporting accuracy (n = 19) was 0.881 ± 0.085 (mean ± SD). The average AUC, reported in 17 studies, was found to be 0.916 ± 0.052, while average sensitivity (n = 16) and specificity (n = 15) were 0.868 ± 0.123 and 0.843 ± 0.235, respectively (Figure 4). A subgroup analysis of AUC (mean ± SD) of classifiers modeled on conventional MRI only (0.907 ± 0.061) vs. advanced MRI (0.930 ± 0.053) did not reach statistical significance (*p* = 0.437) and is represented in Figure S5. Among the 26 best performing classifiers, SVM reached the highest mean (± SD) AUC of 0.936 (±0.045). Mean AUC from the best classifiers grouped by different algorithm types (SVM, DL algorithms, neural networks, tree-based algorithms, and logistic regression) did not vary significantly at an alpha level of 0.05 and are displayed in Figure S4b. Note that these numbers do not represent the results from a meta-analysis, as variance estimates were scarcely reported, and, thus, should be appraised critically. Caution against inference to real-world data is strongly advised.

Ensemble learning approaches were described in three studies. Dong et al. [25] presented an ensemble learning approach, where different traditional ML algorithms were combined for execution of a classification task. The ensemble classifier yielded a classification accuracy of 0.64, tying with the single best performing individual algorithm, a Naïve Bayes classifier, indicating that ensembling was unsuccessful. The same study also proposed an agreement pattern model, a voting ensemble, where only cases with unanimous class labels across all five different classifiers were analysed. This approach achieved a classification accuracy beyond 90%, significantly outperforming the other approaches—however, it was not stated in how many cases total agreement could be achieved. This approach likely favours easy to differentiate cases, thus bearing limited applicability in clinical practice, particularly for more ambiguous studies. Samani et al. [40] presented a similar voting ensemble method. The 2D CNN algorithm performed 16 × 16 voxel patch-wise classifications after training on about 6000 peritumoral patches from 113 training subjects. Training and cross-validation were also conducted patch-wise. Prediction of tumor type at patient level was performed in a holdout test set via majority voting of the individual subordinate patches per patient. While classification accuracy in patch-wise cross validation was 0.85, patient-wise classification after majority voting reached an accuracy of 0.93 in a holdout test set. Shin et al. [36], reporting on a 2D CNN, proceeded similarly, providing cross-validated predictions for 6617 axial sectional images, and concluding patient-wise predictions by majority voting. This validated model achieved an AUC of 0.889 on an internal holdout test set and 0.835 on an external validation set. Splitting imaging data from a subject into further divisions for model training, can serve as means of data augmentation to combat overfitting, a common phenomenon when DL is applied to small datasets.

Two studies reported externally validated classification results. Shin et al. [36] reported a convolutional neural network trained on T1CE- and T2-weighted masks with an AUC of 0.835 (95% CI 0.755–0.915). Bae et al. [22] reported a deep neural network trained on radiomics features retrieved from T1CE and peritumoral T2-weighted masks with an AUC of 0.956 (95% CI 0.918–0.990).

### 3.3. Meta-Analysis

Five studies were found to be eligible for a quantitative meta-analysis of effect estimates. The purpose of this analysis was to obtain robust assembled results on the performance of these ML classifiers. Three studies that reported internally validated AUC values, and two studies reporting externally validated AUC values were analyzed separately in a random effects model. In the internally validated models, an overall AUC of 0.913 (95% CI 0.902–0.925) was attained. Higgins I^2^-test yielded a heterogeneity of 0.00%, however statistical significance (*p* = 0.597) was not given. The meta-analysis from two externally validated models reached an overall AUC of 0.907 (95% CI 0.826–0.988). Heterogeneity, again measured by Higgins I^2^, was at a level of 86.32% at a significance level of *p* < 0.01. This indicates a high level of heterogeneity in the meta-analysis of externally validated classifiers. Forest plots of the meta-analyses are provided in the Supplementary Materials (Figure S2).

### 3.4. Quality of Reporting

Adherence to 29 TRIPOD items was assessed for each study in agreement of two reviewers. These included all TRIPOD items applicable to model development studies according to the official TRIPOD review guidelines, except for item 11 (risk groups), which was not applicable to any included study. Median TRIPOD adherence score was found to be 0.48, reflecting that 14 out of 29 TRIPOD items were fulfilled. TRIPOD adherence scores ranged from 0.17 (5/29) to 0.79 (23/29). The studies published in or after 2019 achieved higher TRIPOD scores on average (*p* = 0.017), with a median of 0.55 (95% CI: 0.49–0.61). On the other hand, the studies published before 2019 had a median TRIPOD score of 0.45 (95% CI: 0.40–0.49).

**Figure 5:**
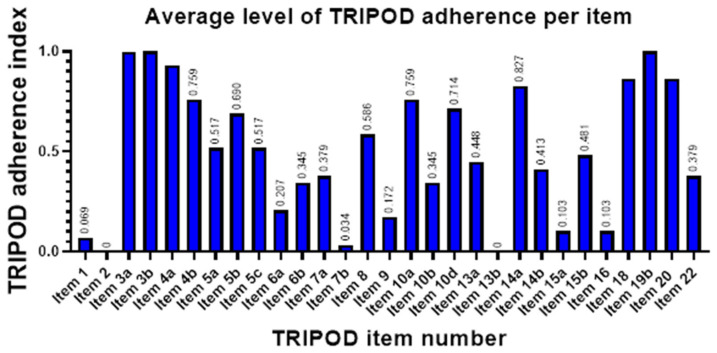
Bar graph of TRIPOD adherence index, a measure for degree of satisfaction for each TRIPOD item applicable in model development studies. Item 11 (Risk groups) was applicable in none of the studies at hand. Item 21 (Supplementary information) is not shown, as it is not included in overall scoring according to official guidelines. Item labels are as follows: 1—Title, 2—Abstract, 3a—Background, 3b—Objectives, 4a—Source of data (Study design), 4b—Source of data (Study dates), 5a—Participants (Study setting), 5b—Eligibility criteria, 5c—Participants (Treatments received), 6a—Outcome (Definition), 6b—Outcome (Blind assessment, 7a—Predictors (Definition), 7b—Predictors (Blind assessment), 8—Sample size, 9—Missing data, 10a—Statistical analysis (Predictors), 10b—Statistical analysis (Model development), 10d—Statistical analysis (Model evaluation), 13a—Flow of participants, 13b—Participant characteristics, 14a—Number of participants and outcomes, 14b—Model development (Predictor and outcome association), 15a—Full model specification, 15b—Model explanation, 16—Model performance, 18—Limitations, 19b—Interpretations, 20—Implications, 22—Funding. Note that items 15a (Full model specification) and 16 (Model performance) are among the TRIPOD items with lowest adherence in the surveyed studies despite having a paramount role for model reproducibility and successful translation to the clinic.

Across studies, average satisfaction per item was measured by what we labeled as adherence index (ADI), with values ranging from zero to one. Highest item satisfaction was detected for Background and Objectives (ADI of 96.1 and 100%, respectively), Study design (93.1%), Model development—Participants and outcomes (82.7%), and Limitations and Implications (both 86.2%). Lowest ADI, indicating low adherence to TRIPOD, were seen in the reporting of Title and Abstract (6.9 and 0%, respectively), Predictors—Blind assessment (3.4%), Participant characteristics (0%), and Full model specification and Model performance (both 10.3%) (Figure 5).

Outside of the framework of TRIPOD, we investigated the reporting on data availability. While six studies [14,32,34,37,38,40] explicitly mentioned data availability upon request, among all twenty-nine examined studies, only Liu et al. [39] provided the algorithm code and radiomics data on an open-source platform.

## 4. Discussion

Previous reports and systematic reviews have corroborated the potential benefit of machine learning for various applications in neuro-oncology, for instance, in prediction of tumor grade, molecular status, or differentiation of glioma from primary central nervous system lymphomas. Predictive ML models for the differentiation of gliomas from brain metastases have the potential to accurately and non-invasively provide preoperative diagnosis and, thus, to influence the strategy for individualized treatment. However, to our knowledge, no study has systematically reviewed the use of machine learning for this differential classification task yet.

Our systematic review was performed under PRISMA guidelines after a thorough search of four databases at three timepoints between September 2020 and September 2021, which resulted in the evaluation of 12,470 abstracts. We extracted information from 29 studies that reported the development of predictive ML models for the differentiation of glioma from brain metastasis.

Our study showed that most articles investigated SVM as classification algorithms and that SVM performed consistently well. As a traditional ML approach, it is a common algorithm in predictive modeling in neuroradiology, due to its simplicity and flexibility [45]. DL algorithms, such as CNN, were described in fewer publications that were more recent. These studies exhibited higher average sample sizes and reported external validation in two out of four instances. Bae et al. presented a deep neural network that outperformed classical ML algorithms, such as AdaBoost or SVM, with an externally validated AUC of 0.956. This aligns with previous studies, which have reported superior results from DL-based algorithms when compared to classical ML techniques for related classification or segmentation tasks [46,47]. DL is widely believed to be one of the major recent advances in the field of machine learning, largely thanks to the increasing availability of big data and growing computational capacities. DL algorithms have been leveraged for different tasks in neuro-oncology, such as acquisition, segmentation, and classification [48]. When compared to classical ML algorithms, which require extraction of handcrafted features, deep neural networks bear the ability to automatically extract relevant features for classification, a process referred to as representation learning [49]. While some studies suggest that DL can outperform classical ML techniques even in small datasets of 50 subjects [50], generally larger datasets are needed to account for the high number of weights within the complex architecture of deep neural networks [51]. As DL algorithms are prone to overfitting in small datasets, which is often the case for medical imaging datasets, validation in external datasets is even more important to preclude over-sensitization to institutional biases. If data is scarce, measures, such as transfer learning [52] or data augmentation should be explored to increase model robustness. Despite promising classification results in our reviewed DL studies, thus, caution is advised, as large, annotated, and high-quality datasets are necessary to prevent overfitting [53]. Ensemble learning methods were trialed in three studies [25,36,40]. Ensemble learning is based on the idea of combining multiple algorithms that were each either trained on different datasets (data diversity) or trained on the same data with differences in algorithm architecture (structural diversity), to generate a model that outperforms the individual classifiers [54]. The final outcome can be obtained either by mathematical fusion of the classifiers or by summing the individually predicted class labels via ensemble voting. This approach can be used to overcome typical challenges in ML, such as overfitting, and to mitigate phenomena, such as class imbalance or the “curse of dimensionality” [55]. Furthermore, we found that the average AUC from the best performing classifiers trained on conventional MRI sequences alone and those integrating information from advanced MRI did not differ significantly. However, we remark that the displayed outcome measures stemmed from internal validation, and hence do not allow inference and generalization to the whole population. In curated datasets, performance metrics are inherently dependent on different factors, such as number of training subjects or data quality that were reported heterogeneously across the displayed studies. This analysis could be repeated once there are enough studies that address this and perform external validation.

Different feature types were used for the classification between gliomas and brain metastases. Three studies compared classification performance using intra- versus peritumoral features and found that the latter achieved higher discrimination performance. This is in line with previously published findings: relative cerebral blood volume (rCBV) measurement has been shown to be a strong discriminator for glioma versus brain metastasis in the peritumoral edema [56,57]. Lu et al. [58] also showed the predictive utility of mean diffusivity from DTI in the peritumoral compartment for distinction between glioma and brain metastasis. Future research should further validate these results in machine learning studies.

Our findings show that there are several limitations that reduce overall model reproducibility, hence posing a barrier for clinical implementation. Most studies relied on single-center datasets, and validation on external imaging datasets was only performed in two studies. This is common in the ML literature on brain tumors because the generation of comprehensive annotated imaging datasets and data sharing are major limitations in the field [59]. Additionally, sample sizes were found to be consistently low at 152.3 ± 144.9. One of the problems of using small datasets is overfitting, where the model captures noise and inherent structures within the training data, which has important ramifications for model generalizability on heterogeneous and unseen data and can overestimate the accuracy measures of algorithms [60]. While there is no consensus on what sample size is too low for application of ML [61], several approaches to determine the minimally required sample size have been proposed [62]. Moreover, several collaborative initiatives, for instance the COINSTAC [63] platform or federated learning (FL) [64], have been developed with the aim of combatting the paucity of large and annotated datasets. FL is an emerging collaborative approach that encompasses multiple centers that train a machine learning model on their institutional private data, and subsequently integrates all model updates into a consensus model. Thereby, FL has been shown to perform comparably to conventional open interinstitutional data sharing models, while bypassing data privacy and confidentiality issues [65].

Another limitation of these studies is the deployment of imbalanced datasets. Classification accuracy was the most frequently provided performance metric in our systematic review. Four studies exclusively provided accuracy as the evaluation metric for their model [28,29,33,66]. However, classification accuracy fails to reliably estimate discriminatory power in the presence of class imbalance, as the impact of a class on model prediction depends on its representation within the data used in the training process [67]. A bias towards the majority class is introduced, as there are fewer cases in the minority class to contribute to overall classification accuracy. Thus, specific characteristics of the minority class are likely to be misidentified as noise and ignored during modeling [68]. Class imbalance can and should be mitigated using various approaches, such as data augmentation, resampling, or employment of penalized models.

All studies used internal validation techniques to evaluate model robustness. Cross-validation techniques, that were most frequently devised for internal validation, iteratively partition the dataset into training and validation sets and are particularly useful when data is scarce, by enabling the exploitation of the entire dataset for modeling. Holdout validation, dividing the samples into designated train and test sets, can, if not stratified, introduce bias when observations are unevenly distributed among training and testing sets, and should typically be reserved for larger datasets. Two studies [31,38] presented a three-way split of their dataset into training, validation, and testing sets. In this setting, the validation set takes on the task of hyperparameter tuning and is typically necessary in complex models with many hyperparameters. Nested cross-validation, described by three studies [35,37,38], addresses the same question of mathematical optimization of the model. To warrant that the models generalize well on unseen data, the choice of internal validation measures should be adapted to the underlying data and individual model and must be reported accordingly.

Several studies included MR scans from brain metastasis patients with multiple foci or failed to explicitly report on the inclusion of solitary BM patients. Recognizing this question is vital when aiming to provide tools for reliable discrimination of brain metastases from GBM, as clinically relevant diagnostic challenges occur in the differentiation of solitary metastases from higher-grade gliomas.

In this systematic review, we provided pooled summary results for the best performing classifiers from each study. Due to a broad heterogeneity among the studies and failure to report necessary effect estimates (AUC) and their variances, not all of them could be subjected to a quantitative meta-analysis. Only small subsets of three and two studies, respectively, were eligible for the conduct thereof. Consistency of effects across the studies is a prerequisite for generalizability in meta-analyses [44]. Further subsampling, as advised for exploration of the cause and type of heterogeneity in our meta-analyses, could not be performed due to the limited number of studies included in the analysis. Overall, our meta-analyses indicate a tendency of the classification performance of eligible studies, all published between 2019 and 2021. However, the limited number of eligible studies indicates the prematurity of this analysis and the need for further validation of these findings. Once the literature fulfils the necessary requirements for the conduct of a more comprehensive meta-analysis, future research could potentially include subgroup analyses, such as comparison of prediction models exploiting information from the intratumoral vs. the peritumoral region, to provide more robust evidence on the effect estimates.

To assess the quality of reporting, we performed a systematic analysis according to the TRIPOD statement. Overall, quality of reporting in the studies was poor. An overall median TRIPOD score of 0.48 (range: 0.17–0.79) signifies that, on average, more than half of the critical information for study development was not reported. Strikingly, our findings are to a large extent in keeping with a recently published systematic review investigating TRIPOD adherence in clinical prediction models in oncology using ML [69]: Dhiman et al. also found low adherence for Title, Abstract. and Predictor blinding; reporting on Background/Objectives and overall interpretation of study results was similarly high in our study cohort; major differences could be seen in the reporting of missing data, where our reviewed articles showed significantly lower adherence; however, given that Dhiman et al. investigated clinical prediction models, it is likely that their results do not translate immediately to ML models based on imaging. This persistent concern of insufficient reporting in the literature necessitates initiatives for data sharing and improvement of transparency.

Based on the deficiencies in current reporting that we identified in our study, we formulated the following recommendations. We propose that authors clearly specify the proportion of solitary brain metastases versus multiple brain metastases in their datasets. The differentiation of solitary brain metastasis from high grade gliomas represents a clinically relevant problem that can be assisted by ML algorithms. On the contrary, differentiation of multiple metastases and solitary metastases from gliomas can have important implications for algorithms that screen studies from normal to abnormal. We advise the use of multi-center hospital datasets for algorithm training and validation. For reporting of model performance, we suggest including multiple performance metrics and statistical testing. Validation of studies in clinically applicable, representative, and independent datasets is crucial for the accurate estimation of generalizability. We understand that such databases may not be readily available; therefore, clear indication of the methods used for validation is critical for future research. Based on our TRIPOD adherence assessment, we recommend providing more descriptive titles that describe the model tested, improving the discussion of results and methods within the abstract, and including balanced datasets with equal representation of tumor types for initial model development. When algorithms are developed for specifically imbalanced dataset applications, then the clear description of the different entities within the dataset is important. We advise authors to sufficiently characterize the predictors used for modeling and to explicitly mention the absence and presence of missing data, respectively. Backed by recent reviews and editorials, we stipulate that strict adherence to standardized reporting guidelines leads to more transparency and can ultimately facilitate model translation and to clinical practice [10,11].

There are several limitations to our study. It is possible that we did not identify every relevant article in the field. To address this limitation, we used four bibliographical databases, as recommended by the Cochrane Handbook for the conduct of systematic reviews. The search was conducted by two institutional librarians and was repeated two times, most recently in September 2021. Another potential limitation is the exclusion of “abstracts only” studies from our systematic review. We acknowledge that this decision, aimed to warrant the inclusion of peer-reviewed results only, could come at the expense of missing pertinent or even contesting evidence to our findings. We prioritized the highest quality of studies over preliminary reports published at scientific meetings. Furthermore, we emphasize that TRIPOD is a quality assessment tool that is tailored for regression-based multivariate prediction models [42], instead of ML techniques that can pursue a different approach for classification. While TRIPOD still provides a rigorous evaluation of ML methods similar to checklists with a focus on AI, such as CLAIM [70], the language in TRIPOD is focused on multivariate regression models. Hence, we acknowledge that translation of TRIPOD to ML studies can be effortful and imprecise. We endorse the development of the TRIPOD–AI extension for explicit use in ML studies, which is currently under development [71].

## 5. Conclusions

We show that the literature demonstrates early evidence for the efficacy of ML algorithms for glioma versus BM classification and paves the way for clinical implementation of potential algorithms. Significant limitations include small datasets, imbalanced representation of pathologies, and lack of external validation of algorithms. This necessitates initiatives for data or algorithm sharing and development of representative multi-center datasets that allow individualization of algorithms to patient populations and imaging protocols from different institutions.

## Figures and Tables

**Table 1:** Overview of study characteristics and best performing classifier from each study. Abbreviations: GBM = Glioblastoma; MET = Brain metastasis; PCNSL = Primary central nervous system lymphoma; MEN = Meningioma; MED = Medulloblastoma; CV = Cross-validation; LOOCV = Leave-One-Out cross-validation; ML = Machine learning; DL = Deep learning; T1CE = contrast-enhanced T1-weighted sequence; DWI = Diffusion weighted imaging; DTI = Diffusion tensor imaging; PWI = Perfusion weighted imaging; rCBV = relative cerebral blood volume; FLAIR = Fluid-attenuated inversion recovery; TE = Time to echo; AUC = Area under the receiver operating characteristic curve; ADC = Apparent diffusion coefficient; LASSO = Least absolute shrinkage and selection operator; SVM = Support vector machine; MLP = Multilayer perceptron; NNW = Neural networks; LogReg = Logistic Regression; DNN = Deep neural network; LDA = Linear discriminant analysis; NB = Naïve Bayes; VFI = Voting feature intervals; KNN = k-nearest neighbors; PNN = Probabilistic neural networks; RF = Random Forest; RBF = Radial basis function kernel; n/a = not available.

Paper	Total Patient Number	Number of Glioma Patients	Number of BM Patients	Ratio of Glioma/met Patients	Solitary BM Only	GBM Only	Tumor Types Studied	Number of Additional Tumors	Number of Patients (Training)	Number of Patients (Validation)	Testing	External Validation	Source of Data	ML Method	Algorithms Used for Classification	Gold Standard for Accuracy	Extracted Feature Types	Best Performing Classifier
Swinburne et al., 2019 [13]	26	9	9	1.000	no	yes	GBM vs. MET vs. PCNSL	8 (PCSNL)	LOOCV			no	single-center	ML	SVM, MLP	Pathology	Perfusion	MLP (Ktrans on T1CE mask) Accuracy: 83.3% AUC: 0.83
Park et al., 2020 [14]	276	137	59	2.322	no	yes	GBM vs. MET vs. PCNSL	80 (PCSNL)	216 (109 GBM, 58 PCNSL, 49 MET)	60 (28 GBM, 22 CNSL, 10 MET)		no	multi-center	DL	CNN	Pathology	Perfusion (Temporal Patterns of Time-Signal Intensity Curves from DSC)	CNN (DSC, FLAIR, T1CE)—internally validated AUC: 0.95 Sensitivity: 0.9 Specificity: 0.857
Shrot et al., 2019 [15]	141	41	38	1.079	no	yes	GBM vs. MET vs. PCNSL vs. MEN	12 (PCSNL), 50 (Meningioma)	LOOCV			no	single-center	ML	Decision tree (SVM)	Pathology	Morphology, Diffusion, Perfusion	Binary hierarchical tree with SVM classifier (T1, T1c, T2, FLAIR, DTI, DSC) Sensitivity: 0.974 Specificity: 0.969
Yamashita et al., 2008 [16]	126	95	19	5.000	multiple	no	Glioma vs. MET vs. PCNSL	12 (PCSNL)	LOOCV			no	not specified	ML	3-layered NNW	Pathology	Clinical, Qualitative/Semantic imaging features	ANN AUC: 0.946 board-certified radiologists without ANN: Accuracy: 87.9% AUC: 0.923 Sensitivity: 0.808 Specificity: 0.903 board-certified radiologists with ANN: Accuracy: 91.5% AUC: 0.946 Sensitivity: 0.868 Specificity: 0.931
Blanchet et al., 2011 [17]	33	18	15	1.200	solitaty	yes	GBM vs. MET		LOOCV			no	single-center	ML	k-means clustering	Pathology	Shape	k-means clustering (T1, T2) Accuracy: 93.9%
Tsolaki et al., 2013 [18]	49	35	14	2.500	solitary	yes	GBM vs. MET		10-fold CV			no	single-center	ML	SVM, Naive Bayes, KNN	Pathology	Spectroscopy	SVM (MRS: NAA; rCBV)—peritumoral Accuracy: 98% Sensitivity: 0.98 Specificity: 0.99 SVM (MRS: NAA/Cr; rCBV)—intratumoral Accuracy: 95% Sensitivity: 0.94 Specificity: 0.95
Yang et al., 2014 [19]	48	30	18	1.667	solitary	yes	GBM vs. MET		LOOCV			no	single-center	ML	QDA, NB, SVM, KNN, NNW (MLP architecture)	Pathology	Shape, Diffusion	Neural Network (DTI) Accuracy: 97.92% AUC: 0.975 Sensitivity: 100% Specificity: 96.55%
Tateishi et al., 2020 [20]	127	73	53	1.377	multiple, largest selected for classification	yes	GBM vs. MET		5-fold CV			no	single-center	ML	SVM	Pathology, clinical history of path-proven primary cancer	Texture	SVM (T1CE, T2, ADC) AUC: 0.92
Abidin et al., 2019 [21]	52	35	17	2.059	solitary	yes	GBM vs. MET		stratified 10-fold CV			no	single-center	ML	AdaBoost	Pathology	First-order statistics, Texture, Higher-order-features: Topology (Minkowski functionals), Wavelet-transformed, Local Binary Patterns (LBP)	AdaBoost (Local Binary Pattern, T1CE) AUC: 0.846
Bae et al., 2020 [22]	248	159	89	1.787	solitary	yes	GBM vs. MET		166 (109 GBM, 57 MET)	82 (50 GBM, 32 MET)		yes	single-center	ML and DL	DNN, AdaBoost, (L-SVM, LDA)	Pathology	DL extracted (DL) Shape, First-order statistics, Texture (traditional ML)	Deep Neural Network (T1CE)—internal AUC: 0.986 Deep Neural Network (T1CE)—external AUC: 0.956 Accuracy: 89% Sensitivity: 0.906 pecificity: 0.88
Artzi et al., 2019 [23]	439	212	227	0.934	solitary	yes	GBM vs. MET vs. MET-subtypes		5-fold CV			no	single-center	ML	SVM, KNN, decision trees, ensemble classifiers	Pathology	Clinical features, Qualitative/semantic imaging features, Morphology, First-order statistics, Texture, Higher-order features: Wavelet features, Bagof-features	SVM (T1CE) Accuracy: 89% AUC: 0.96 Sensitivity: 0.86 Specificity: 0.85
Yang et al., 2016 [24]	48	30	18	1.667	solitary	yes	GBM vs. MET		LOOCV			no	single-center	ML	SVM	Pathology	Shape	SVM (DTI, Cluster 1 & 4) Accuracy: 95.83% AUC: 0.983 Sensitivity: 0.9444 Specificity: 0.9667
Dong et al., 2020 [25]	120	60	60	1.000	solitary	n/a	Glioma vs. MET		84 (42 GBM, 42 MET)	36 (18 GBM, 18 MET)		no	single-center	ML	NNW, DT, NB, KNN, SVM	Radiological	Shape, First-order statistics, Texture	Naive Bayes (T1, T1CE, T2) Accuracy: 60% Sensitivity: 0.45 Specificity: 0.75 Combined(LOG) [Decision Tree, SVM, NNW, kNN, NB] Accuracy: 64% Sensitivity: 0.5 Specificity: 0.73 Agreement of all 5 classifier: Accuracy: 94% Sensitivity: 1 Specificity: 0.89
Meier et al., 2020 [26]	109	25	84	0.298	231 lesions in 109 patients	yes	GBM vs. MET		stratified 3-fold CV			no	single-center	ML	SVM	Pathology	Qualitative/Semantic imaging features	SVM (Qualitative image features) F1-Score: 0.865
Georgiadis et al., 2008 [27]	67	21	19	1.105	no	no	Glioma vs. MET vs. MEN	27 (Meningioma)	external cross-validation (ECV) with 3-fold split			no	single-center	ML	PNN, LSFT-PNN, SVM-RBF, ANN, Cubic LSFT-PNN, Quardratic LSFT-PNN	Radiological	Texture	ANN (T1)—Primary tumors vs. Secondary tumors (MET + Meningioma) Accuracy: 100%
Tsolaki et al., 2015 [28]	126	80	22	3.636	solitary	no	Glioma vs. MET vs. MEN	24 (Meningioma)	10-fold cross validation			no	single-center	ML	SVM, Naïve Bayes, k-NN, LDA	Pathology	Spectroscopy, Diffusion, Perfusion	SVM (DWI/DTI/PWI/short TE)—peritumoral Accuracy: 98% SVM (DWI/DTI/PWI/short TE)—intratumoral Accuracy: 96%
Zacharaki et al., 2009 [27]	98	74	24	3.083	no	no	Glioma vs. MET vs. MEN	4 (Meningioma)	LOOCV			no	single-center	ML	SVM, k-NN, LDA	Pathology	Shape, First-order statistics, Texture	SVM (FLAIR, T2, T1ce, rCBV, T1) Accuracy: 84.7% AUC: 0.882 Sensitivity: 0.882 Specificity: 0.865
Zacharaki et al., 2011 [29]	97	73	23	3.174	no	no	Glioma vs. MET vs. MEN		LOOCV			no	single-center	ML	VFI, KNN, Naive Bayes	Pathology	Clinical, Shape, First-order	kNN with wrapper evaluator Accuracy: 96.91%
Svolos et al., 2013 [30]	115	73	18	4.056	solitary	no	Glioma vs. MET vs. MEN	24 (atypical Meningioma)	10-fold cross validation			no	single-center	ML	SVM	Pathology	Diffusion, Perfusion	SVM (HGG Grade 4 vs. MET) (ADC, FA, rCBV)—peritumoral Accuracy: 96% Sensitivity: 0.98 Specificity: 0.94
Sachdeva et al., 2016 [31]	428	177	66	2.682	no	no	Glioma vs. MET vs. MEN vs. MED	97 (Meningioma), 88 (Medulloblastoma)	40% training, 10% testing, 50% validation	40% training, 10% testing, 50% validation	40% training, 10% testing, 50% validation	no	public dataset (PGIMER and SPL datasets)	ML	GA, GA-SVM, GA-ANN	Radiological	First-order statistics, Texture	GA-ANN—no binary classification Accuracy: 94% (imputed)
Payabvash et al., 2020 [32]	248	99	65	1.523	no	no	Glioma vs. MET vs. MED vs. Hemangioblastoma vs. Ependymoma	Hemangioblastoma (n = 44), Ependymoma (n = 27), Medulloblastoma (n = 26).	10-fold cross validaiton			no	single center	ML	NB, RF, NN, SVM	Pathology	Clinical (Age), Qualitative/Semantic imaging features, Diffusion	Random Forest—MET vs. All primary tumors Accuracy: 83% AUC: 0.82 Sensitivity: 55.6 Specificity: 92.6 PPV: 73.9
Qin et al., 2019 [33]	42	24	18	1.333	solitary	yes	GBM vs. MET		5-fold cross validation			no	single center	ML	Decision trees, LDA, LogReg, linear SVM, KNN	Pathology	First-order, Second-order (Energy)	kNN Accuracy: 92.9%
Chen et al., 2019 [34]	134	n/a	n/a		no	yes	GBM vs. MET		80%	20%		no	single center	ML	LDA, SVM, RF, KNN, Gaussian NB	Pathology	Texture	LogReg + Distance correlation Accuracy: 79% AUC: 0.8 Sensitivity: 0.8 Specificity: 0.71
Ortiz-Ramón et al., 2020 [35]	100	50	50	1.000	no	yes	GBM vs. MET		nested cross-validation			no	single center	ML	random forest (RF), support vector machine (SVM) with linear kernel, k-nearest neighbors (KNN), naïve Bayes (NB) and multilayer perceptron (MLP)	Radiological	Texture	MLP Accuracy: 81% AUC: 0.91 Sensitivity: 0.91 Specificity: 0.8
Shin et al., 2021 [36]	741	482	259	1.861	solitary	yes	GBM vs. MET		450	48	100	143	multi-center	DL	CNN (2D)	Pathology	DL extracted	CNN (2D; T1CE, T2)—internal Accuracy: 89% AUC: 0.889 Sensitivity: 0.939 Precision: 0.852 CNN—external Accuracy: 85.9% AUC: 0.835 Sensitivity: 0.889 Precision: 0.907
Priya et al., 2021 [37]	120	60	60	1.000	no	yes	GBM vs. MET		nested cross-validation			no	single center	ML	Linear (LASSO, Elastic Net) and logistic regression, NNW, SVM- MLP, RF, AdaBoost	Clinico-Radiological	Shape, First-order statistics, Texture	LASSO (T1, T1CE, T2, FLAIR, ADC) Accuracy: 89.2% AUC: 0.953 Sensitivity: 0.887 Specificity: 0.897
de Causans et al., 2021 [38]	180	92	88	1.045	multiple, largest selected for classification	yes	GBM vs. MET		143 (71 GBM, 72 BM)	nested cross-validation (10 repeated 5-fold CV)	37 (21, 16)	no	multi-center	ML	LogReg (Yeo-Johnson scaling features)	Pathology	Shape, First-order statistics, Texture	LogReg (T1CE) Accuracy: 80% Sensitivity: 0.75 Specificity: 0.86
Liu et al., 2021 [39]	268	140	128	1.094	solitary	yes	GBM vs. MET		208 (110 GBM, 98 BM)	10-fold cross validation	60 (30, 30)	no	single center	ML	RF, DT, LogReg, AdaBoost, Gaussian processing, SVM	Pathology	Shape, First-order statistics, Texture, Higher-order: Wavelet-transformed, Laplace of Gaussian	Random Forest (Boruta selection) (T1CE) Accuracy: 85% AUC: 0.97 Sensitivity: 0.84 Specificity: 0.93
Samani et al., 2021 [40]	136	86	50	1.720	no, 3 patients with multifocal metastasis	yes	GBM vs. MET		108 (66 GBM, 40 BM)	5-fold cross validation	30 (20, 10)	no	single center	DL	2D CNN	Pathology	Diffusion	CNN (2D, DTI, FW-VP map)—patch wise Accuracy: 85% AUC: 0.9 Sensitivity: 0.87 Specificity: 0.81 CNN—majority vote, subject-wise: Accuracy: 93%

## Data Availability

Data is contained within the article or supplementary material.

## Supplementary Materials

The following supporting information can be downloaded at: https://www.mdpi.com/article/10.3390/cancers14061369/s1, Figure S1: Search strategy used on bibliographic databases; Figure S2: Meta-analysis results from random effect models displayed as forest plots; Figure S3: Types of imaging features across all studies; Figure S4: (a) Number of times that algorithm types were reported in a study vs. algorithm representation among the best classifiers extracted from each study; (b) AUC of the best reported classifiers grouped by type of algorithm leveraged for classification; Figure S5: AUC of the best reported classifiers grouped by type of imaging modality leveraged for classification.
