# Supplementary material for: Machine Learning Applications for Differentiation of Glioma from Brain Metastasis—A Systematic Review
Source: Cancers (Basel). 2022 Mar 8;14(6):1369. doi: 10.3390/cancers14061369 (PMC8946855; doi:10.3390/cancers14061369)
Supplement: Supplementary file 1 [file cancers-14-01369-s001.zip › cancers-1582611-supplementary.pdf]

cancers

Supplementary Materials

Machine Learning Applications for Differentiation of Glioma from Brain Metastasis—A Systematic Review

Leon Jekel [1,2,3], Waverly R. Brim [1,4], Marc von Reppert [1], Lawrence Staib [1], Gabriel Cassinelli Petersen [1], Sara Merkaj [1], Harry Subramanian [1], Tal Zeevi [1], Seyedmehdi Payabvash [1], Khaled Bousabarah [5], MingDe Lin [1,6], Jin Cui [1], Alexandria Brackett [7], Amit Mahajan [1], Antonio Omuro [8], Michele H. Johnson [1], Veronica L. Chiang [9,10], Ajay Malhotra [1], Björn Scheffler [2,3] and Mariam S. Aboian [1,*]

Embase <1974 to 2021 September 08>
https://ovidsp.ovid.com/ovidweb.cgi?T=JS&NEWS=N&PAGE=main&SHAREDSEARCHID=6WWZM06axM5bXayO-KOp2GnSlQIudbVkrWT5p6EDlSCauSnw2Y6khzIzrcsfP0b0ty

1 exp Artificial Intelligence/ 51643
2 machine learning/ 45594
3 deep learning/ 18019
4 ((artificial* or machine* or deep*) adj3 (intelligence or learning)).tw,kw. 93681
5 AI.ti,ab. 42888
6 exp computer assisted diagnosis/ 1227573
7 computer* assist* diagnosis.tw,kw. 980
8 radiomics/ 3291
9 radiomic*.tw,kw. 6431
10 or/1-9 1386577
11 exp nuclear magnetic resonance imaging/ 1052392
12 (Magnetic Resonance Imag* or MR-Imag* or MR Imag or MRI* or NMR).tw,kw. 918522
13 11 or 12 1337174
14 exp glioma/ 146867
15 glioma*.tw,kw. 88327
16 (glial adj2 (tumor* or tumour*)).tw,kw. 3708
17 (glioblastoma* or astrocytoma* or astrocytic glioma* or astroglioma).tw,kw. 80831
18 or/14-17 173080
19 10 and 13 and 18 10286
20 limit 19 to yr="2021 - 2022" 646

Ovid MEDLINE(R) ALL <1946 to September 08, 2021>
https://ovidsp.ovid.com/ovidweb.cgi?T=JS&NEWS=N&PAGE=main&SHAREDSEARCHID=6WWZM06axM5bXayO-KOp2GmxDv3He5vMs34mXuyB8r3xetSEdenzouB8SBVkR16O7d

1 exp Artificial Intelligence/ 122439
2 ((artificial* or machine* or deep*) adj3 (intelligence or learning)).tw,kw. 70443
3 AI.ti,ab. 31484
4 exp Image Interpretation, Computer-Assisted/ 567619
5 computer* assist* diagnosis.tw,kw. 665
6 radiomic*.tw,kw. 4310
7 or/1-6 744339
8 exp Magnetic Resonance Imaging/ 486836
9 (Magnetic Resonance Imag* or MR-Imag* or MR Imag or MRI*).tw,kw. 454635
10 8 or 9 650559
11 exp Glioma/ 89120
12 glioma*.tw,kw. 62652

13 (glial adj2 (tumor or tumour)).tw,kw. 853
14 (glioblastoma* or astrocytoma* or astrocytic glioma* or astroglioma).tw,kw. 54111
15 or/11-14 120216
16 7 and 10 and 15 4436
17 limit 16 to yr="2021 - 2022" 203

Cochrane CENTRAL
#1 MeSH descriptor: [Artificial Intelligence] explode all trees1174
#2 (artificial* OR machine* OR deep*) AND (intelligence OR learning) 3825
#3 AI 8367
#4 MeSH descriptor: [Image Processing, Computer-Assisted] explode all trees3637
#5 computer* assist* diagnosis 6699
#6 radiomic* 324
#7 #1 OR #2 OR #3 OR #4 OR #5 OR #6 22212
#8 MeSH descriptor: [Magnetic Resonance Imaging] explode all trees 8161
#9 Magnetic Resonance Imag* OR MR-Imag* OR MR Imag OR MRI* OR NMR 38512
#10 #8 OR #9 38620
#11 MeSH descriptor: [Glioma] explode all trees 1254
#12 glioma* 1854
#13 (glial AND (tumor OR tumour)) 75
#14 glioblastoma* OR astrocytoma* OR astrocytic glioma* OR astroglioma 2531
#15 #11 OR #12 OR #13 OR #14 3720
#16 #7 AND #10 AND #15 with Publication Year from 2020 to 2021, in Trials 8

Web of Science
https://www.webofscience.com/wos/woscc/summary/10205a39-d5e8-4aff-955c-a938dfac56b8-07a123c0/relevance/1
5
((#1) AND #2) AND #3 and 2021 (Publication Years)
189

4
((#1) AND #2) AND #3
925

3
((TS=(glioma*)) OR TS=(glial NEAR/2 (tumor* or tumour*))) OR TS=(glioblastoma* or astrocytoma* or astrocytic glioma* or astroglioma)
138,478

2
TS=(Magnetic Resonance Imag* or MR-Imag* or MR Imag or MRI* or NMR)
1,088,733

1
((((TS=((artificial* or machine* or deep*) NEAR/3 (intelligence or learning))) OR TI=(AI)) OR AB=(AI)) OR TS=(computer* assist* diagnosis)) OR TS=(radiomic*)
410,154

Embase <1974 to 2021 January 29>
1 exp Artificial Intelligence/ 45007

2 machine learning/ 37007
3 deep learning/ 12393
4 ((artificial* or machine* or deep*) adj3 (intelligence or learning)).tw,kw. 73708
5 AI.ti,ab. 39008
6 exp computer assisted diagnosis/ 1169775
7 computer* assist* diagnosis.tw,kw. 937
8 radiomics/ 1903
9 radiomic*.tw,kw. 4867
10 or/1-9 1305625
11 exp nuclear magnetic resonance imaging/ 1001848
12 (Magnetic Resonance Imag* or MR-Imag* or MR Imag or MRI* or NMR).tw,kw. 882389
13 11 or 12 1278307
14 exp glioma/ 139715
15 glioma*.tw,kw. 84577
16 (glial adj2 (tumor* or tumour*)).tw,kw. 3616
17 (glioblastoma* or astrocytoma* or astrocytic glioma* or astroglioma).tw,kw. 77347
18 or/14-17 165100
19 10 and 13 and 18 9560
20 limit 19 to yr="2020 - 2022" 771

Ovid MEDLINE(R) ALL <1946 to January 29, 2021>

1 exp Artificial Intelligence/ 106412
2 ((artificial* or machine* or deep*) adj3 (intelligence or learning)).tw,kw. 55350
3 AI.ti,ab. 28603
4 exp Image Interpretation, Computer-Assisted/ 551508
5 computer* assist* diagnosis.tw,kw. 626
6 radiomic*.tw,kw. 3204
7 or/1-6 706556
8 exp Magnetic Resonance Imaging/ 465045
9 (Magnetic Resonance Imag* or MR-Imag* or MR Imag or MRI*).tw,kw. 435821
10 8 or 9 625065
11 exp Glioma/ 85314
12 glioma*.tw,kw. 60258
13 (glial adj2 (tumor or tumour)).tw,kw. 831
14 (glioblastoma* or astrocytoma* or astrocytic glioma* or astroglioma).tw,kw. 51784
15 or/11-14 115828
16 7 and 10 and 15 4493
17 limit 16 to yr="2020 - 2021" 260

Cochrane CENTRAL (trials)

ID Search Hits
#1 MeSH descriptor: [Artificial Intelligence] explode all trees 1040
#2 (artificial* OR machine* OR deep*) AND (intelligence OR learning) 3131
#3 AI 7937
#4 MeSH descriptor: [Image Processing, Computer-Assisted] explode all trees 3582
#5 computer* assist* diagnosis 6489
#6 radiomic* 210
#7 #1 OR #2 OR #3 OR #4 OR #5 OR #6 20843
#8 MeSH descriptor: [D008279] explode all trees 0
#9 Magnetic Resonance Imag* OR MR-Imag* OR MR Imag OR MRI* OR NMR 36332

#10 #8 OR #9 36332
#11 MeSH descriptor: [Glioma] explode all trees 1197
#12 glioma* 1792
#13 (glial AND (tumor OR tumour)) 70
#14 glioblastoma* OR astrocytoma* OR astrocytic glioma* OR astroglioma 2432
#15 #11 OR #12 OR #13 OR #14 3580
#16 #7 AND #10 AND #15 with Publication Year from 2020 to 2021, in Trials 2

Web of Science
13
235
#12
Indexes=SCI-EXPANDED, SSCI, A&HCI, CPCI-S, CPCI-SSH, BKCI-S, BKCI-SSH, ESCI, CCR-EXPANDED, IC Timespan=2020-2021

12
711
#11 AND #7 AND #6
Indexes=SCI-EXPANDED, SSCI, A&HCI, CPCI-S, CPCI-SSH, BKCI-S, BKCI-SSH, ESCI, CCR-EXPANDED, IC Timespan=All years

11
132,043
#10 OR #9 OR #8
Indexes=SCI-EXPANDED, SSCI, A&HCI, CPCI-S, CPCI-SSH, BKCI-S, BKCI-SSH, ESCI, CCR-EXPANDED, IC Timespan=All years

10
75,253
TS=(glioblastoma* or astrocytoma* or astrocytic glioma* or astroglioma)
Indexes=SCI-EXPANDED, SSCI, A&HCI, CPCI-S, CPCI-SSH, BKCI-S, BKCI-SSH, ESCI, CCR-EXPANDED, IC Timespan=All years

9
2,783
TS=(glial NEAR/2 (tumor* or tumour*))
Indexes=SCI-EXPANDED, SSCI, A&HCI, CPCI-S, CPCI-SSH, BKCI-S, BKCI-SSH, ESCI, CCR-EXPANDED, IC Timespan=All years

8
91,055
TS=(glioma*)
Indexes=SCI-EXPANDED, SSCI, A&HCI, CPCI-S, CPCI-SSH, BKCI-S, BKCI-SSH, ESCI, CCR-EXPANDED, IC Timespan=All years

7
1,046,719
TS=(Magnetic Resonance Imag* or MR-Imag* or MR Imag or MRI* or NMR)
Indexes=SCI-EXPANDED, SSCI, A&HCI, CPCI-S, CPCI-SSH, BKCI-S, BKCI-SSH, ESCI, CCR-EXPANDED, IC Timespan=All years

6
331,970
#5 OR #4 OR #3 OR #2 OR #1
Indexes=SCI-EXPANDED, SSCI, A&HCI, CPCI-S, CPCI-SSH, BKCI-S, BKCI-SSH, ESCI, CCR-EXPANDED, IC Timespan=All years

5

4,619
TS=(radiomic*)
Indexes=SCI-EXPANDED, SSCI, A&HCI, CPCI-S, CPCI-SSH, BKCI-S, BKCI-SSH, ESCI, CCR-EXPANDED, IC
# 4
6,845
TS=(computer* assist* diagnosis)
Indexes=SCI-EXPANDED, SSCI, A&HCI, CPCI-S, CPCI-SSH, BKCI-S, BKCI-SSH, ESCI, CCR-EXPANDED, IC Timespan=All years

3
51,251
AB=(AI)
Indexes=SCI-EXPANDED, SSCI, A&HCI, CPCI-S, CPCI-SSH, BKCI-S, BKCI-SSH, ESCI, CCR-EXPANDED, IC Timespan=All years

2
11,596
TI=(AI)
Indexes=SCI-EXPANDED, SSCI, A&HCI, CPCI-S, CPCI-SSH, BKCI-S, BKCI-SSH, ESCI, CCR-EXPANDED, IC Timespan=All years

1
276,118
TS=((artificial* or machine* or deep*) NEAR/3 (intelligence or learning))
Indexes=SCI-EXPANDED, SSCI, A&HCI, CPCI-S, CPCI-SSH, BKCI-S, BKCI-SSH, ESCI, CCR-EXPANDED, IC Timespan=All years
Search Log_Glioma MRI AI
09/21/2020
Alexandria Brackett

Database: Embase <1974 to 2020 September 18>
Search Strategy:
----
1 exp Artificial Intelligence/ (41398)
2 machine learning/ (32735)
3 deep learning/ (9556)
4 ((artificial* or machine* or deep*) adj3 (intelligence or learning)).tw,kw. (63111)
5 AI.ti,ab. (36883)
6 exp computer assisted diagnosis/ (1134120)
7 computer* assist* diagnosis.tw,kw. (897)
8 radiomics/ (1317)
9 radiomic*.tw,kw. (4166)
10 or/1-9 (1257205)
11 exp nuclear magnetic resonance imaging/ (973321)
12 (Magnetic Resonance Imag* or MR-Imag* or MR Imag or MRI* or NMR).tw,kw. (858203)
13 11 or 12 (1243003)
14 exp glioma/ (136239)
15 glioma*.tw,kw. (82490)
16 (glial adj2 (tumor* or tumour*)).tw,kw. (3562)
17 (glioblastoma* or astrocytoma* or astrocytic glioma* or astroglioma).tw,kw. (75319)
18 or/14-17 (160999)
19 10 and 13 and 18 (9266)

Database: Ovid MEDLINE(R) ALL <1946 to September 18, 2020>
Search Strategy:

----
1 exp Artificial Intelligence/ (99921)
2 ((artificial* or machine* or deep*) adj3 (intelligence or learning)).tw,kw. (45705)
3 AI.ti,ab. (26932)
4 exp Image Interpretation, Computer-Assisted/ (541841)
5 computer* assist* diagnosis.tw,kw. (591)
6 radiomic*.tw,kw. (2606)
7 or/1-6 (684173)
8 exp Magnetic Resonance Imaging/ (455739)
9 (Magnetic Resonance Imag* or MR-Imag* or MR Imag or MRI* or NMR).tw,kw. (595729)
10 8 or 9 (780376)
11 exp Glioma/ (83794)
12 glioma*.tw,kw. (58224)
13 (glial adj2 (tumor or tumour)).tw,kw. (816)
14 (glioblastoma* or astrocytoma* or astrocytic glioma* or astroglioma).tw,kw. (49925)
15 or/11-14 (112476)
16 7 and 10 and 15 (4381)

Cochrane trials (CENTRAL)
ID Search Hits
#1 MeSH descriptor: [Artificial Intelligence] explode all trees990
#2 (artificial* OR machine* OR deep*) AND (intelligence OR learning) 2659
#3 AI 7619
#4 MeSH descriptor: [Image Interpretation, Computer-Assisted] explode all trees 7481
#5 computer* assist* diagnosis 6352
#6 radiomic* 153
#7 #1 OR #2 OR #3 OR #4 OR #5 OR #6 23662
#8 MeSH descriptor: [Magnetic Resonance Imaging] explode all trees 7660
#9 Magnetic Resonance Imag* OR MR-Imag* OR MR Imag OR MRI* OR NMR 34562
#10 #8 OR #9 34661
#11 MeSH descriptor: [Glioma] explode all trees 1169
#12 glioma* 1725
#13 (glial AND (tumor OR tumour)) 67
#14 glioblastoma* OR astrocytoma* OR astrocytic glioma* OR astroglioma 2328
#15 #11 OR #12 OR #13 OR #14 3430
#16 #7 AND #10 AND #15 in Trials 83

Web of Science
Search History

12
619
#11 AND #7 AND #6
Indexes=SCI-EXPANDED, SSCI, A&HCI, CPCI-S, CPCI-SSH, BKCI-S, BKCI-SSH, ESCI, CCR-EXPANDED, IC

11
128,127
#10 OR #9 OR #8
Indexes=SCI-EXPANDED, SSCI, A&HCI, CPCI-S, CPCI-SSH, BKCI-S, BKCI-SSH, ESCI, CCR-EXPANDED, IC Timespan=All years

10

72,878
TS=(glioblastoma* or astrocytoma* or astrocytic glioma* or astroglioma)
Indexes=SCI-EXPANDED, SSCI, A&HCI, CPCI-S, CPCI-SSH, BKCI-S, BKCI-SSH, ESCI, CCR-EXPANDED, IC Timespan=All years

9
2,729
TS=(glial NEAR/2 (tumor* or tumour*))
Indexes=SCI-EXPANDED, SSCI, A&HCI, CPCI-S, CPCI-SSH, BKCI-S, BKCI-SSH, ESCI, CCR-EXPANDED, IC Timespan=All years

8
88,517
TS=(glioma*)
Indexes=SCI-EXPANDED, SSCI, A&HCI, CPCI-S, CPCI-SSH, BKCI-S, BKCI-SSH, ESCI, CCR-EXPANDED, IC Timespan=All years

7
1,023,935
TS=(Magnetic Resonance Imag* or MR-Imag* or MR Imag or MRI* or NMR)
Indexes=SCI-EXPANDED, SSCI, A&HCI, CPCI-S, CPCI-SSH, BKCI-S, BKCI-SSH, ESCI, CCR-EXPANDED, IC Timespan=All years

6
298,785
#5 OR #4 OR #3 OR #2 OR #1
Indexes=SCI-EXPANDED, SSCI, A&HCI, CPCI-S, CPCI-SSH, BKCI-S, BKCI-SSH, ESCI, CCR-EXPANDED, IC Timespan=All years

5
3,866
TS=(radiomic*)
Indexes=SCI-EXPANDED, SSCI, A&HCI, CPCI-S, CPCI-SSH, BKCI-S, BKCI-SSH, ESCI, CCR-EXPANDED, IC Timespan=All years

4
6,591
TS=(computer* assist* diagnosis)
Indexes=SCI-EXPANDED, SSCI, A&HCI, CPCI-S, CPCI-SSH, BKCI-S, BKCI-SSH, ESCI, CCR-EXPANDED, IC Timespan=All years

3
48,357
AB=(AI)
Indexes=SCI-EXPANDED, SSCI, A&HCI, CPCI-S, CPCI-SSH, BKCI-S, BKCI-SSH, ESCI, CCR-EXPANDED, IC Timespan=All years

2
10,849
TI=(AI)
Indexes=SCI-EXPANDED, SSCI, A&HCI, CPCI-S, CPCI-SSH, BKCI-S, BKCI-SSH, ESCI, CCR-EXPANDED, IC Timespan=All years

1
244,818
TS=((artificial* or machine* or deep*) NEAR/3 (intelligence or learning))
Indexes=SCI-EXPANDED, SSCI, A&HCI, CPCI-S, CPCI-SSH, BKCI-S, BKCI-SSH, ESCI, CCR-EXPANDED, IC Timespan=All years

Figure S1: Search strategy used on bibliographic databases.4 databases were searched at three different timepoints (September 18, 2020; January 29, 2021; September 8, 2021).

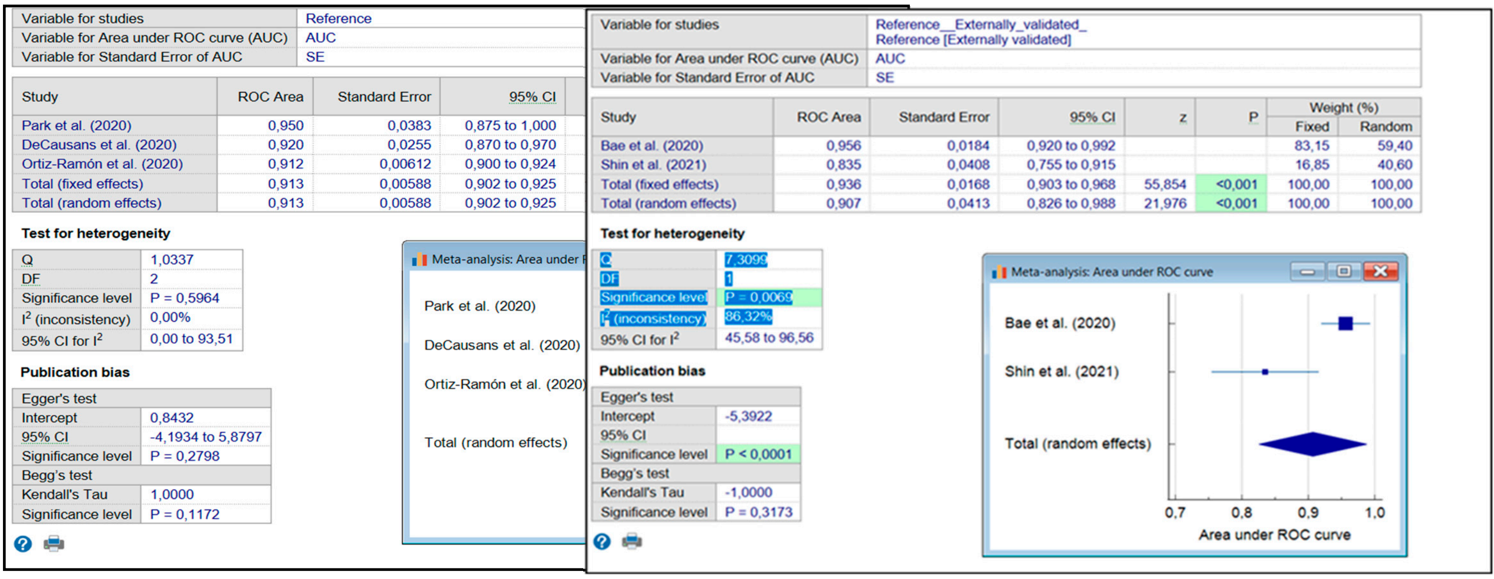

Figure S2. Meta-analysis results from random effect models displayed as forest plots. This figure shows the results from the meta-analyses of all eligible internally validated (panel on the left-hand side) and externally validated (panel on the right-hand side) studies. The forest plots show the calculated average means (box) and their according 95% confidence interval (error bars).

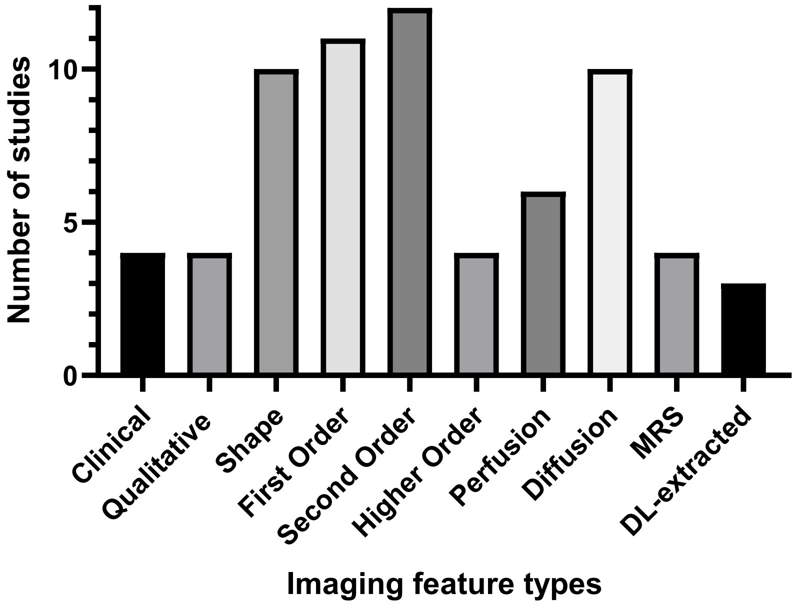

Figure S3. Types of imaging features across all studies. This bar graph illustrates the number of studies reporting use of certain imaging features. Imaging features were clustered as clinical features (e.g. age, gender); qualitative imaging features (e.g. tumor location or VASARI features); first-order, i.e. intensity-based, radiomics features; second-order, i.e. textural, radiomics features; higher-order, such as wavelet transformed, radiomics features; features from perfusion MRI; diffusion MRI; MR spectroscopy; and deep-learning extracted imaging features. Second-order (textural) quantitative imaging features were leveraged most frequently (n=12).

a

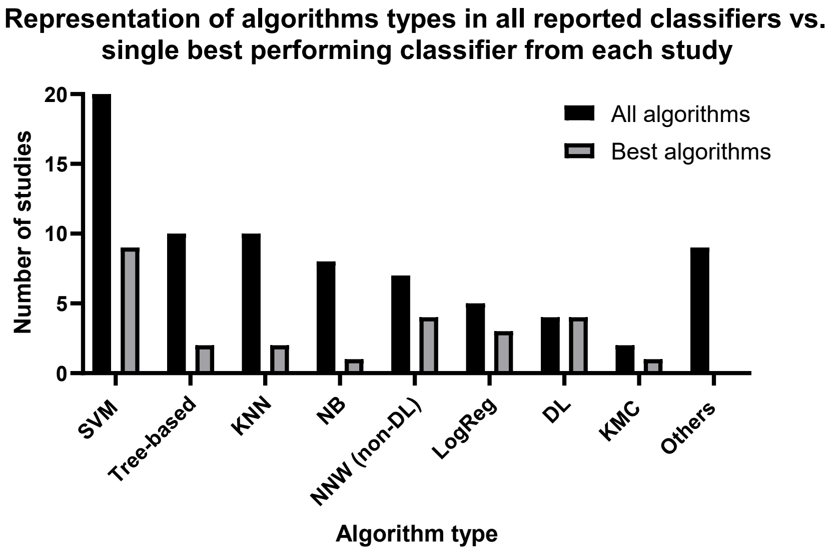

b

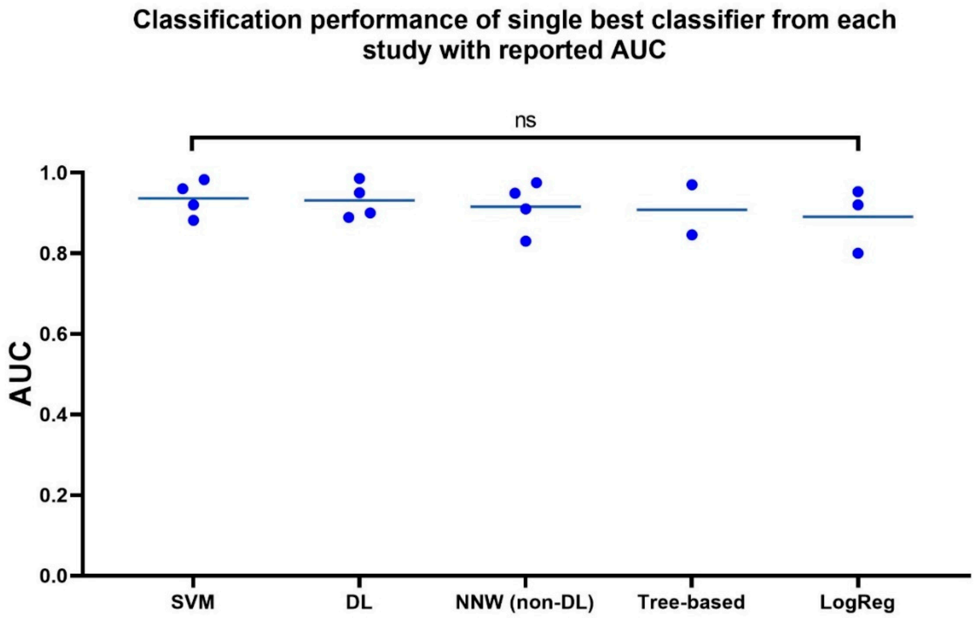

Figure S4 (a) Number of times algorithm types were reported in a study vs. algorithm representation among the best classifiers extracted from each study (n=26). This grouped bar graph illustrates the representation of algorithm types among all reported classifiers (black) and the representation thereof among the best performing classifiers (grey). Note that support vector machines (SVM) were reported in most (n=20) studies, and, similarly, returned best classification results in the highest number of studies (n=9). KNN = k-nearest neighbors; NB = Naïve Bayes; NNW = Neural Networks; DL = deep learning; LogReg = Logistic Regression; KMC = k-means clustering.(**b)** AUC of best reported classifiers grouped by type of algorithm leveraged for classification. Only studies that reported the AUC (n=17) were considered, thus the displayed data points do not entirely accumulate to the numbers illustrated in bar graph S4a. The difference in mean AUC between the different algorithm types did not reach statistical significance (alpha level = 0.05) from Student's t-test. However, keep in mind that outcome measures, such as AUC, are inherently related to the number of training subjects, degree of data curation and quality, and method of validation, which were reported very heterogeneously among all studies. Ns = not significant; SVM = support vector machine; DL = deep learning; NNW = neural networks; LogReg = Logistic Regression.

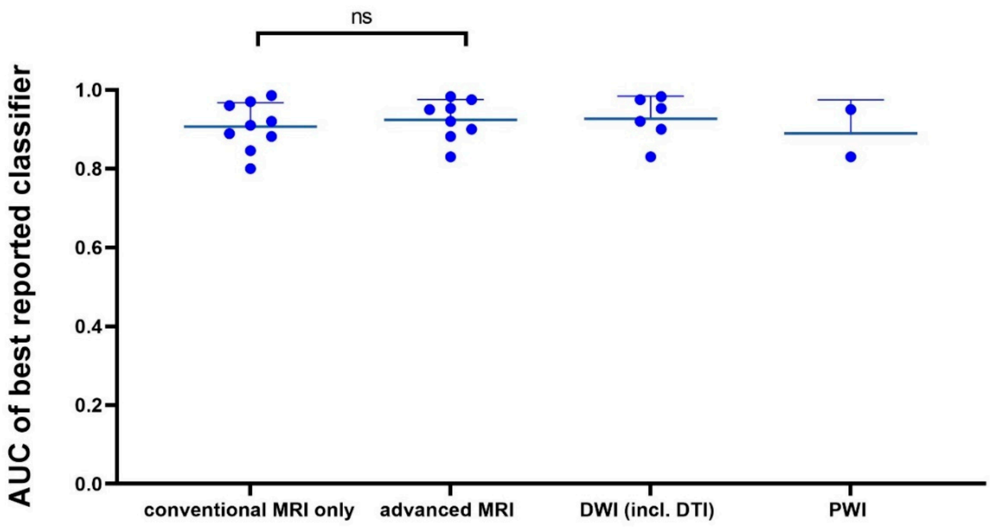

Figure S5. AUC of best reported classifiers grouped by type of imaging modality leveraged in the study. Only studies that reported the AUC (n=17) are displayed. Student's t-test did not return statistical significance for difference between mean AUC in the conventional MRI only and advanced MRI study cohorts. Note that outcome measures, such as AUC, are inherently related to the number of training subjects, degree of data curation and quality, and method of validation, which were reported very heterogeneously among all studies. Ns = not significant.
